# Pharmacokinetic interaction between regorafenib and atorvastatin in rats

**DOI:** 10.1007/s43440-024-00570-z

**Published:** 2024-04-18

**Authors:** Danuta Szkutnik-Fiedler, Edyta Szałek, Filip Otto, Andrzej Czyrski, Marta Karaźniewicz-Łada, Anna Wolc, Edmund Grześkowiak, Konrad Lewandowski, Agnieszka Karbownik

**Affiliations:** 1 Department of Clinical Pharmacy and Biopharmacy, Poznań University of Medical Sciences, Rokietnicka 3, 60-806 Poznań, Poland; 2 Department of Physical Pharmacy and Pharmacokinetics, Poznań University of Medical Sciences, Rokietnicka 3, 60-806 Poznań, Poland; 3 Department of Animal Science, Iowa State University, 239E Kildee Hall, Ames, IA 50011 USA; 4 Hy-Line International, 2583 240th Street, Dallas Center, IA 50063 USA

**Keywords:** Regorafenib, Atorvastatin, Pharmacokinetics, Drug–drug interaction

## Abstract

**Background:**

Regorafenib is used in the treatment of colorectal cancer and hepatocellular carcinoma. Due to the co-morbidity of hyperlipidemia in these conditions, statins, including atorvastatin, are used as potential adjuvant therapy agents. Both regorafenib and atorvastatin are metabolized by CYP3A4. In addition, atorvastatin is a P-gp and BCRP substrate, whereas regorafenib and its active metabolites M-2 and M-5 are inhibitors of these transporters. Hence, the concomitant use of both drugs may increase the risk of a clinically significant drug–drug interaction. Therefore, the present study aimed to assess the pharmacokinetic interactions of atorvastatin and regorafenib and their active metabolites.

**Methods:**

Male Wistar rats were assigned to three groups (eight animals in each) and were orally administered: regorafenib and atorvastatin (I_REG+ATO_), a carrier with regorafenib (II_REG_), and atorvastatin with a carrier (III_ATO_). Blood samples were collected for 72 h. UPLC-MS/MS was the method of measurement of regorafenib and atorvastatin concentrations. The pharmacokinetic parameters were calculated with a non-compartmental model.

**Results:**

A single administration of atorvastatin increased the exposure to regorafenib and its active metabolites. In the I_REG+ATO_ group, the *C*_max_, AUC_0–*t*_, and AUC_0–∞_ of regorafenib increased 2.7, 3.2, and 3.2-fold, respectively. Atorvastatin also significantly increased the *C*_max_, AUC_0–*t*_, and AUC_0–∞_ of both regorafenib metabolites. Regorafenib, in turn, decreased the AUC_0–*t*_ and AUC_0–∞_ of 2-OH atorvastatin by 86.9% and 67.3%, and the same parameters of 4-OH atorvastatin by 45.0% and 46.8%, respectively.

**Conclusions:**

This animal model study showed a significant pharmacokinetic interaction between regorafenib and atorvastatin. While this interaction may be clinically significant, this needs to be confirmed in clinical trials involving cancer patients.

**Graphical abstract:**

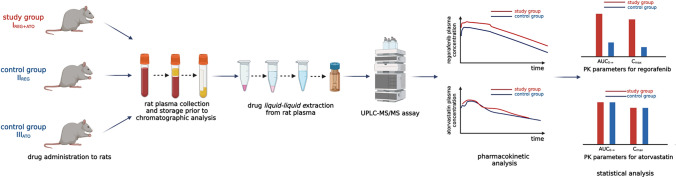

## Introduction

Colorectal cancer (CRC) is the most common malignancy worldwide, among both men and women [1]. Nearly 25% of CRC patients present metastases at the time of diagnosis [2] and about half develop metastases during cancer progression, which significantly increases mortality risk [1, 3]. Moreover, a growing number of CRC cases are diagnosed in patients under the age of 50 [1].

Regorafenib (REG) is a drug commonly used for CRC treatment [4]. REG targets multiple protein kinases, including those involved in tumor angiogenesis, oncogenesis, and metastases [5, 6]. It also blocks the activity of vascular endothelial growth factor (VEGF), angiopoietin (TIE2), fibroblast growth factor receptor (FGFR), as well as the platelet-derived growth factor receptor (PDGFR), and the rearranged during transfection (RET) oncogene. Furthermore, REG inhibits the mutated KIT kinase, an important factor stimulating oncogenesis in gastrointestinal stromal tumors (GIST) [3, 5–7]. During REG therapy, attention should be paid to the possibility of interactions with other drugs, especially at the metabolic stage. In humans, REG is primarily metabolized in the liver through cytochrome P450 3A4 (CYP3A4)-mediated oxidation and UGT1A9-mediated glucuronidation. M-2 (regorafenib *N*-oxide) and M-5 (*N*-desmethyl regorafenib N-oxide) are the main circulating metabolites of REG in human plasma. Both of these are pharmacologically active and have similar concentrations to REG at a steady state [3, 5, 6].

Statins, such as atorvastatin (ATO), are among the drugs often combined with REG. As hyperlipidemia increases the risk of CRC, ATO lowers lipid concentration through complete inhibition of 3-hydroxy-3-methylglutaryl-coenzyme A (HMG-CoA) reductase [8]. Moreover, this compound can also inhibit cancer cell proliferation, migration, and survival [9, 10]. The reason for ATO interaction with other drugs also lies in its complex metabolism. ATO metabolism is mainly mediated by the hydroxylation of CYP3A4 to active ortho- (2-OH ATO), parahydroxylated (4-OH ATO), and various beta-oxidative metabolites. Ortho- and parahydroxylated metabolites account for 70% of instances of systemic inhibition HMG-CoA reductase activity. The orthohydroxy metabolite is further metabolized by glucuronidation. In addition to being a CYP3A4 substrate, ATO may inhibit also this isoenzyme in vitro [11].

REG, its active metabolites (M-2 and M-5), and ATO, are also substrates of P-glycoprotein (P-gp) and BCRP (Breast Cancer Resistance Protein), resulting in their inhibition (P-gp is inhibited by both and BCRP only by REG) [5, 6, 11]. Considering the metabolism of both drugs, it cannot be excluded that their simultaneous use could increase the risk of clinically significant drug–drug interactions. Therefore, our study aimed to assess the bilateral pharmacokinetic interactions of ATO and REG and their active metabolites in rats. In preclinical studies, male rats are widely used as an animal model, due to the relatively similar to human activity of CYP3A.

## Materials and methods

### Chemicals and reagents

REG (CAS No. 755037-03-7), M-2, M-5, and REG-d3 (internal standard for the quantification of REG as well as M-2 and M-5) were purchased from LGC Standards (Łomianki, Poland). ATO as a calcium salt trihydrate (CAS No. 344423-98-9), ortho- and parahydroxylated ATO (as dihydrate monosodium and disodium salts, respectively) were purchased from Santa Cruz Biotechnology (Dallas, USA). Methanol, acetonitrile (liquid chromatography and mass spectroscopy grade), rosuvastatin calcium salt (internal standard for the quantification of both ATO and its metabolites), ammonium formate, ammonium acetate, and dimethyl sulfoxide (DMSO) were purchased from Sigma-Aldrich (Saint Louis, Missouri, USA). The water used in the mobile phase was deionized, distilled, and filtered through a Millipore system (Direct Q3, Millipore, USA) before use. REG (Stivarga^®^, batch No. BXJRJJ1) was purchased from Bayer Poland Sp. z o.o (Warsaw, Poland). ATO (Atoris, batch No. DA5950) was purchased from KRKA-Polska Sp. z o.o. (Warsaw, Poland).

### Animals and study design

The study was conducted on healthy, well-fed 14-week-old adult male Wistar rats (*n* = 24). The animals were fed a standard diet with free access to fresh water. All required international, national, and/or institutional guidelines on the care and use of animals were followed. The experiments were carried out according to the European Union Directive 2010/63/EU and approved by the Local Ethics Committee (located in the Department of Animal Physiology and Biochemistry, Poznań University of Life Sciences, Wołyńska 35, 60-637 Poznań, Poland; approval no. 32/2022 of 27.05.2022).

Three groups of animals (mean weight, 439.5 ± 43.6 g) were used in the study: controls, II_REG_ (*n* = 8) and III_ATO_ (*n* = 8), received REG and ATO, respectively, along with 1 mL of drug carrier; the study group, I_REG+ATO_ (*n* = 8), received REG with ATO. Following the decision of the Local Ethics Committee, the pharmacokinetic data from the control group (III_ATO_), receiving ATO alone, were adopted from a previous project on pharmacokinetic interactions between sorafenib and ATO, and sorafenib and metformin [12]. This reduced the number of animals included in the experiment, which adheres to the assumptions of the 3R concept. However, the adoption of the results was significantly limited due to administration of drugs (same manufacturer, same dose) from different batches. Nonetheless, no other experimental conditions were changed. REG [20 mg/kg body weight (b.w.)] [6] and ATO (20 mg/kg b.w.) [13] were administered using a gastric probe (1 mL of each solution) directly into the stomachs of live animals. Solutions of REG and ATO were prepared in 10% dimethyl sulfoxide (DMSO) and 0.9% sodium chloride, respectively. Blood samples were collected from the tail vein before the administration of the drugs, and at the following time points: 0.25, 0.5, 1, 2, 3, 5, 7, 9, 24, 26, 48, and 72 h for REG; 0.25, 0.5, 0.75, 1, 2, 4, 6, and 12 h for ATO and the control group (III_ATO_); and 0.25, 0.5, 1, 2, 3, 5, 7, and 9 h for the I_REG+ATO_ group. The plasma was separated from blood through centrifugation (2880 g, 10 min at 4 °C). Plasma samples were kept at − 80 °C until analysis.

### ATO and REG quantification in blood plasma using UPLC-MS/MS

The concentrations of both drugs and their metabolites in the animals’ plasma were analyzed using the UPLC MS/MS methods [14].

The analytical methods were validated according to the European Medicines Agency guidelines [15]. For the quantification of ATO, 2-OH ATO, and 4-OH ATO, the same UPLC-MS/MS analytical conditions were maintained as in the control group (III_ATO_).

### UPLC-MS/MS analysis of REG, M-2, and M-5

The following extraction procedure was applied: 50 μL of internal standard (200 µg/mL REG d-3 solution) was added to 20 μL of rat plasma and mixed for 10 s. Next, 20 μL of acetonitrile was added. The samples were mixed for 10 min and then centrifuged for 10 min at 8500 rpm. 50 μL of the resulting supernatant was filtered and used for further analysis. Each sample was analyzed twice, and the arithmetic mean was determined from the surface areas obtained in the chromatogram.

Stock solutions, at a concentration of 10 mg/mL REG, M-2, and M-5, were prepared by dissolving 10 mg of the respective compounds in 1 mL DMSO. Working solutions, used afterwards to prepare standard solutions, were prepared by diluting the stock solutions with methanol (MeOH) to obtain 100 000, 10 000, 1000, and 100 ng/mL concentrations.

The internal standard solution was prepared by diluting the initial standard of REG-d3 (1 mg of the compound was dissolved in 1 mL DMSO) with MeOH, reaching a concentration of 200 ng/mL.

The LC–MS/MS system consisted of a Xevo TQ-S-micro triple quadrupole mass spectrometer coupled with a UPLC Acquity I-class PLUS (Waters Corporation, Milford, MA, USA), and the Waters Software MassLynx V4.2 SCN1017 Software. A Cortecs UPLC C18 column (2.1 × 50 mm, 1.6 μm) was equipped with an Acquity UPLC BEH C18 VanGuard pre-column (2.1 × 5 mm, 3/Pk, Waters Corporation). The injection volume was 5 μL. The temperature of the autosampler was 4 °C, and the temperature of the column was 40 °C. Mobile phase A was composed of 0.1% aqueous formic acid solution (v/v), and phase B of acetonitrile: methanol and 0.1% formic acid aqueous solution at 1:3 (v/v) ratio. The total run time was 5 min. The gradient was as follows: 0–0.5 min, with 10% B; 0.5–3 min 95% B; 3–4 min, 95% B; 4–4.1 min, 10% B; 4.1–5 min, 10% B. The flow rate was 0.4 mL/min. Positive electrospray ionization (ESI) multiple reaction monitoring (MRM) experiments were conducted for sample analysis. The Xevo TQ-S Micro Mass spectrometer (Waters) was run in the positive ion mode, and configured in the multiple reaction monitoring mode, for the detection of REG, REG-*N*-oxide (M-2), *N*-desmethyl-REG-*N*-oxide (M-5), and isotope-labelled REG-d3.

The data were collected and processed using the MassLynx Software (V 4.2 SCN 1017, Waters). The following settings were used for the Xevo TQ-S Micro Mass spectrometer: source temperature—150 °C, desolvation temperature—600 °C, nitrogen gas flow—900 L/h, capillary voltage 3.6 kV. Transition ion pairs (parent *m/z*—daughter *m/z*) were identified, using the MRM mode, for the following compounds: 482.95–270.08 and 288.02 for REG; 499.00–304.01, 252.16, and 229.00 for M-2; 485.94–202.02 and 228.98 for M-5; and 486.02–273.07 for REG-d3 as an IS.

### UPLC-MS/MS analysis of ATO, 2-OH ATO, and 4-OH ATO

Briefly, 20 μL of the rosuvastatin solution (an internal standard), at a concentration of 200 ng/mL, was added to 20 μL of rat plasma. Then, 100 μL of the ammonium acetate buffer (pH 4.6) was added, and the samples were subjected to liquid–liquid extraction with 1000 μL of ethyl acetate. After shaking and centrifuging for 10 min each, the organic layer was transferred to the concentration tubes and evaporated at 45 °C under vacuum (Concentrator plus, Eppendorf, USA). The dry residue was dissolved with a 50 μL of water-acetonitrile (50:50, v/v) solution containing 0.1% formic acid, and a volume of 20 μL was injected directly into the UPLC–MS/MS system.

Stock solutions ATO (0.5 mg/mL), 2-OH ATO (0.1 mg/mL), 4-OH ATO (0.1 mg/mL), and rosuvastatin (0.5 mg/mL) were prepared by dissolving 5.41 mg of ATO, 1.10 mg of 2-OH ATO, 1.08 mg of 4-OH ATO, and 5.20 mg of rosuvastatin salts in 10 mL of acetonitrile each. Then, the working solutions were prepared by diluting the stock solutions with acetonitrile to obtain 10 mg/L concentration. Working solutions were used subsequently to prepare standard solutions.

Chromatographic separation was performed using a Shimadzu UPLC Nexera set (Shimadzu Co., Kyoto, Japan) equipped with a five-channel degasser (DGU-20A5) and a thermostated autosampler (SIL-30AC). The Zorbax Eclipse Plus C18 column (2.1 mm × 100 mm, 3.5 µm) (Agilent Technologies, USA), thermostated at 40 °C, was used as the stationary phase. The mobile phase was a mixture of deionized water (A) and acetonitrile (B), both containing 0.1% (v/v) formic acid. The gradient was as follows: 0–2 min linear, from 50 to 70% B; 2–4 min 70% B; 4–6 min return, from 70 to 50% B; and a post-time of 4 min with 50% B for column equilibration. The mobile phase was delivered at a 0.3 mL/min flow rate. Positive electrospray ionization mode (ESI+) was applied for eluent introduction from the UPLC column to the MS interface. The electrospray needle voltage was maintained at 3.5 kV. The MS interface had the following parameters: desolvation line 250 °C, heat block temperature 400 °C, interface temperature 350 °C. The drying gas and the nebulizing gas flow rates were 12 and 2 L/min, respectively. Ion transitions of the tested compounds were observed in the multiple reaction monitoring (MRM) mode, and their identification was based on the ratio of the mass of the obtained ions to their charges (*m/z*). For the quantitative analysis, the most intense precursor-to-product mass transition was selected: *m/z* 558.8–440.1 for ATO, 547.9–440.2 for 2-OH and 4-OH ATO, and 481.7–258.0 for rosuvastatin.

### Pharmacokinetic assay

WinNonlin (Certara, USA) was used to calculate the following pharmacokinetic parameters: the area under the concentration–time curve from zero to the last measurable concentration (AUC_0–*t*_), the area under the plasma concentration–time curve from zero to infinity (AUC_0–∞_), the absorption rate constant (*k*_a_), the elimination rate constant (*k*_e_), the elimination half-life (*t*_1/2_), the apparent plasma drug clearance (Cl/F), and the apparent volume of distribution (*V*_d_/*F*). The maximum plasma concentration (*C*_max_) and the time to reach the *C*_max_ (*t*_max_) were obtained directly from the measured values. Non-compartmental analysis (NCA) with linear interpolation was used to calculate the values of the pharmacokinetic parameters.

All these parameters were subjected to statistical analysis.

### Statistical analysis

The SAS software version 9.4 (SAS Institute Inc., Cary, NC 27513, USA) was used for statistical analysis. The Shapiro–Wilk test was used to test for normality. Two pairs of groups were independently analyzed: I_REG+ATO_ vs. II_REG_ and I_REG+ATO_ vs. III_ATO._ The differences between the normally distributed variables were determined using the Student’s *t*-test. Non-normally distributed variables were analyzed using the Mann–Whitney test. A *p*-value of < 0.05 was considered significant.

## Results

### Analytical methods

Precision and accuracy for REG, ATO, and their major metabolites are presented in Table 1. The validation procedure followed the latest guidelines established by the European Medicines Agency (ICH guidelines M10 on bioanalytical method validation—Step 5; date of first publication 27 July 2022 [15]).

**Table 1:** Precision and accuracy for regorafenib, atorvastatin and their major metabolites

Drug/drug metabolite	Precision	Accuracy	Calibration curve range (ng/mL)	*r*	*R*^2^
	Intra- and inter-assay (LLOQ) (%)				
REG	(< 16.2) < 15.1	(< 12.0) < 11.4	50–8000	0.997	0.998
M-2	(< 14.8) < 14.2	(< 9.9) < 9.3	10–2500	0.997	0.997
M-5	(< 13.2) < 12.0	(< 13.0) < 12.5	1–175	0.997	0.997
ATO	(< 18.7) < 12.2	(< 14.0) < 13.8	0.2–200	0.999	0.998
2-OH ATO	(< 18.7) < 13.1	(< 10.2) < 10.0	0.2–200	0.999	0.997
4-OH ATO	(< 14.0) < 12.1	(< 6.1) < 6.0	0.2–200	0.998	0.998

*REG* regorafenib, *ATO* atorvastatin, *M-2* regorafenib *N*-oxide, *M-5* N-desmethyl regorafenib *N*-oxide, *2-OH* *ATO* orthohydroxy atorvastatin, 4*-OH ATO* parahydroxy atorvastatin, *LLOQ* lower limit of quantification, *r* correlation coefficient, *R*^*2*^ coefficient of determination

### The influence of ATO on the pharmacokinetics of REG and M-2 and M-5

The results of statistical analysis are presented in Table 2.

**Table 2:** The plasma pharmacokinetic parameters of REG and its metabolites M-2 and M-5 after the oral administration of a single dose of REG (20 mg/kg b.w.) to the II_REG_ group and REG + ATO (20 mg/kg b.w. + 20 mg/kg b.w.) to the I_REG+ATO_ group

Pharmacokinetic parameters	II_REG_ (*n* = 8)	I_REG+ATO_ (*n* = 8)	*p*-value I_REG+ATO_ *vs* II_REG_	*G*_mean_ ratio* (90% CI) I_REG+ATO_ vs II_REG_
*REG*				
*C*_max_ (µg/mL)	1.88 (1.66; 2.02)	7.29 (6.24; 7.54)	0.0009 *U* = 36, N1 = 8, N2 = 8	3.72 (3.20; 4.32)*
AUC_0–*t*_ (µg × h/mL)	35.82 (34.31; 37.66)	163.18 (131.49; 171.13)	0.0009 *U* = 36, N1 = 8, N2 = 8	4.20 (3.57; 4.95)*
AUC_0–∞_ (µg × h/mL)	35.95 (34.42; 37.72)	163.37 (131.59; 171.50)	0.0009 *U* = 36, N1 = 8, N2 = 8	4.20 (3.56; 4.94)*
*t*_max_ (h)	6.00 (4.00; 7.00)	5.00 (4.00; 5.00)	0.3159 *U* = 77.5, N1 = 8, N2 = 8	0.85 (0.63; 1.17)*
*k*_a_ (h^−1^)	0.41 ± 0.25 (62.4)	0.59 ± 0.17 (29.7)	0.1170 *t*_14_ = 1.67	1.60 (1.09; 2.37)
*k*_el_ (h^−1^)	0.11 ± 0.03 (22.8)	0.10 ± 0.02 (20.0)	0.3629 *t*_14_ = − 0.94	0.91 (0.75; 1.10)
*t*_1/2_ (h)	6.37 ± 1.33 (20.8)	7.02 ± 1.53 (21.8)	0.3774 *t*_14_ = 0.91	1.10 (0.91; 1.33)
Cl/F (L/h × kg)	0.21 (0.21; 0.23)	0.06 (0.05; 0.07)	0.0009 *U* = 100, N1 = 8, N2 = 8	0.26 (0.22; 0.30)*
*V*_d_/*F* (L)	1.98 (1.50; 2.46)	0.51 (0.50; 0.62)	0.0009 *U* = 100, N1 = 8, N2 = 8	0.28 (0.22; 0.37)*
*M-2*				
*C*_max_ (ng/mL)	302.15 (249.90; 395.95)	1647.45 (1374.65; 1994.65)	0.0009 *U* = 36, N1 = 8, N2 = 8	5.03 (3.82; 6.62)*
AUC_0–*t*_ (ng × h/mL)	9548.40 (8488.70; 11,642.31)	52,182.11 (40,840.43; 64,989.90)	0.0009 *U* = 36, N1 = 8, N2 = 8	5.47 (4.30; 6.96)*
AUC_0–∞_ (ng × h/mL)	10,947.48 (9718.99; 12,781.14)	52,438.36 (41,030.58; 65,561.89)	0.0009 *U* = 36, N1 = 8, N2 = 8	4.55 (3.70; 5.60)*
*M-2/REG ratio*				
*C*_max_	0.18 (0.13; 0.28)	0.27 (0.20; 0.33)	0.2271 *U* = 56, N1 = 8, N2 = 8	1.35 (0.60; 3.07)*
AUC_0–*t*_	0.26 (0.20; 0.31)	0.31 (0.27; 0.45)	0.2701 *U* = 57, N1 = 8, N2 = 8	1.30 (0.96; 1.76)*
AUC_0–∞_	0.30 (0.26; 0.38)	0.31 (0.27; 045)	0.8748 *U* = 66, N1 = 8, N2 = 8	1.08 (0.81; 1.46)*
*M-5*				
*C*_max_ (ng/mL)	26.90 (23.40; 28.65)	150.05 (136.75; 155.40)	0.0009 *U* = 36, N1 = 8, N2 = 8	5.65 (5.06; 6.31)*
AUC_0–*t*_ (ng × h/mL)	791.48 (703.15; 884.23)	5501.55 (5310.04; 5967.36)	0.0009 *U* = 36, N1 = 8, N2 = 8	7.13 (6.13; 8.29)*
AUC_0–∞_ (ng × h/mL)	833.10 (752.92; 929.13)	5919.80 (5556.63; 6371.62)	0.0009 *U* = 36, N1 = 8, N2 = 8	7.16 (6.21; 8.26)*
*M-5/REG ratio*				
*C*_max_	0.01 (0.01; 0.02)	0.02 (0.02; 0.02)	0.0181 *U* = 45, N1 = 8, N2 = 8	1.52 (0.68; 3.39)*
AUC_0–*t*_	0.02 ± 0.01 (23.3)	0.04 ± 0.01 (18.3)	0.0002 *t*_14_ = 4.99	1.70 (1.42; 2.03)
AUC_0–∞_	0.02 ± 0.01 (22.8)	0.04 ± 0.01 (18.1)	0.0002 *t*_14_ = 5.13	1.71 (1.43; 2.03)

*REG* regorafenib, *ATO* atorvastatin, *M-2* regorafenib *N*-oxide, *M-5* *N*-desmethyl regorafenib *N*-oxide, *n* number of animals, *CI* confidence interval, *C*_*max*_ the maximum plasma concentration, *AUC*_*0*–*t*_ the area under the plasma concentration–time curve from zero to the time of the last measurable concentration, *AUC*_*0*–∞_ the area under the plasma concentration–time curve from zero to infinity, *t*_*max*_ the time to the first occurrence of *C*_max_, *k*_*el*_ the elimination rate constant, *t*_*1*/*2*_ the elimination half-life, *Cl/F* the apparent plasma drug clearance, *V*_*d*_/*F* the apparent volume of distribution

*Non-parametric test. Arithmetic means and standard deviations (SD) are shown with coefficients of variation CV (%) in brackets for parametric test; median values with quartiles in case of non-parametric test

**Fig. 1:**
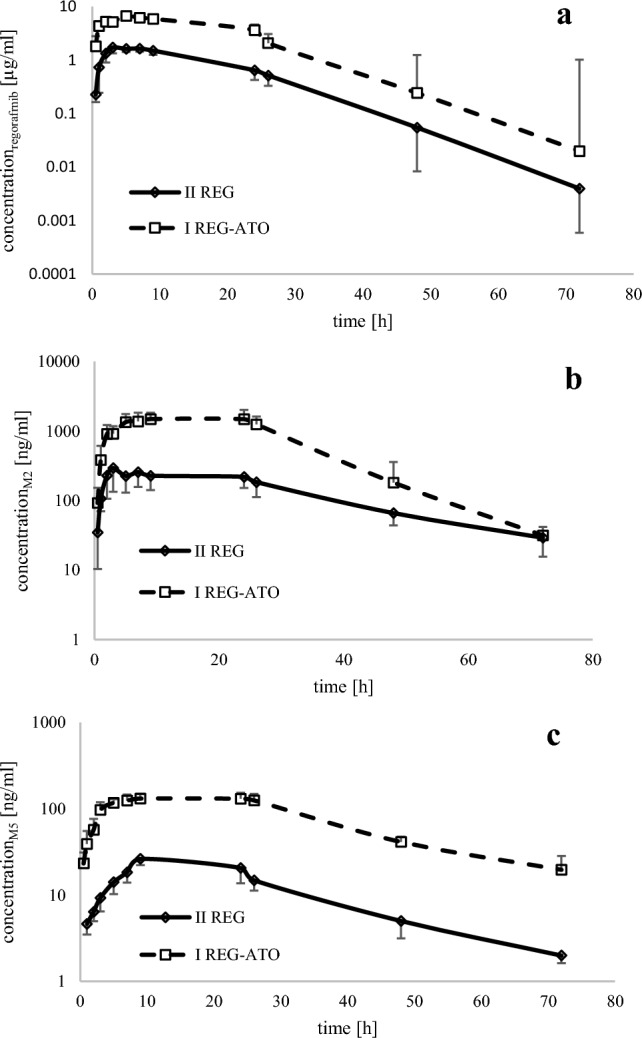
The REG (**a**), M-2 (**b**) and M-5 (**c**) plasma concentration–time profiles in rats after a single oral dose of REG 20 mg/kg b.w. (II_REG_; *n* = 8) and REG 20 mg/kg b.w. + ATO 20 mg/kg b.w. (I_REG + ATO_; *n* = 8). *REG* regorafenib, *ATO* atorvastatin, *M-2* regorafenib *N*-oxide, *M-5* *N*-desmethyl regorafenib *N*-oxide, *n* number of animals

After the administration of REG with ATO (I_REG+ATO_ group), the *C*_max_, AUC_0–*t*_, and AUC_0–∞_ of REG increased 2.7, 3.2, and 3.2-fold, respectively, as compared to the control II_REG_ group (Table 2, Fig. 1a).

In the presence of ATO, Cl/F and *V*_d_/*F* of REG decreased by 72.7% and 71.7%, respectively. No statistically significant differences were found in REG *t*_max_, *k*_a_, *k*_el_, or *t*_0.5_ (Table 2).

After the administration of ATO, the *C*_max_, AUC_0–*t*_, and AUC_0–∞_ of M-2 were, respectively, 3.9, 4.5, and 3.6-fold greater, whereas the values of the same parameters for M-5 were, respectively, 4.6, 6.0, and 6.0-fold greater (Table 2, Fig. 1b, c).

The M-2/REG ratios for the *C*_max,_ AUC_0–*t*_, and AUC_0–∞_ were similar in both groups, whereas in the presence of ATO, the M-5/REG ratios increased by 66.7%, 100.0%, and 100.0%, respectively (Table 2).

The sum of the *C*_max_ values for REG, M-2, and M-5 in the ATO group (8086.43 ± 2698.35 ng/mL; CV = 33.4) was significantly greater (*p* = 0.033; *G*_mean_ ratio = 3.93, and 90% CI = 3.93; 2.44) than in the II_REG_ group (2078.95 ± 691.36 ng/mL; CV = 33.3).

### The influence of REG on the pharmacokinetics of ATO, 2-OH ATO, and 4-OH ATO

The results of statistical analysis are presented in Table 3.

**Table 3:** The plasma pharmacokinetic parameters for ATO, 2-OH ATO, and 4-OH ATO after the oral administration of a single dose of ATO (20 mg/kg b.w.) to the III_ATO_ group and REG + ATO (20 mg/kg b.w. + 20 mg/kg b.w.) to the I_REG+ATO_ group

Pharmacokinetic parameters	III_ATO_ (*n* = 8)	I_REG+ATO_ (*n* = 8)	*p*-value I_REG+ATO_ vs III_ATO_	*G*_mean_ ratio* (90% CI) I_REG+ATO_ vs III_ATO_
*ATO*				
*C*_max_ (ng/mL)	75.12 (47.94; 85.00)	64.18 (50.64; 117.20)	0.8748 U = 66, N1 = 8, N2 = 8	1.10 (0.73; 1.65)*
AUC_0–*t*_ (ng × h/mL)	162.58 (147.07 190.31)	187.00 (125.88; 317.08)	0.8748 *U* = 66, N1 = 8, N2 = 8	1.13 (0.74; 1.74)*
AUC_0–∞_ (ng × h/mL)	169.45 (162.03; 194.68)	196.06 (138.38; 325.75)	0.7929 *U* = 65, N1 = 8, N2 = 8	1.15 (0.77; 1.73)*
*t*_max_ (h)	1.00 (1.00; 2.00)	1.00 (0.75; 2.00)	0.9102 *U* = 69, N1 = 8, N2 = 8	1.05 (0.47; 2.33)*
*k*_el_ (h^−1^)	0.24 ± 0.08 (33.7)	0.38 ± 0.15 (38.4)	0.0355 *t*_14_ = 2.33	1.54 (1.08; 2.20)
*t*_1/2_ (h)	3.22 ± 1.33 (41.4)	2.14 ± 1.01 (47.2)	0.0896 *t*_14_ = − 1.82	0.65 (0.45; 0.93)
Cl/*F* (L/h × kg)	54.31 ± 13.87 (25.5)	46.19 ± 22.22 (48.1)	0.3953 *t*_14_ = − 0.88	0.77 (0.51; 1.18)
*V*_d_/*F* (L)	257.67 ± 132.63 (51.5)	156.60 ± 133.05 (85.0)	0.1503 *t*_14_ = − 1.52	0.50 (0.26; 0.95)
*2-OH ATO*				
*C*_max_ (ng/mL)	126.09 (87.49; 276.16)	82.27 (55.25; 92.10)	0.0933 *U* = 41, N1 = 8, N2 = 7	0.56 (0.31; 1.00)*
AUC_0–*t*_ (ng × h/mL)	544.83 (448.90; 682.46)	38.94 (26.20; 65.78)	0.0015 *U* = 28, N1 = 8, N2 = 7	0.08 (0.04; 0.16)*
AUC_0–∞_ (ng × h/mL)	695.68 ± 297.16 (42.7)	227.66 ± 204.61 (89.9)	0.0039 *t*_13_ = − 3.50	0.20 (0.08; 0.47)
*2-OH ATO/ATO*				
*C*_max_	2.53 (1.27; 3.27)	0.95 (0.60; 1.37)	0.0728 *U* = 40, N1 = 8, N2 = 7	0.49 (0.28; 0.86)*
AUC_0–*t*_	3.14 (2.70; 3.81)	0.27 (0.07; 0.35)	0.0015 *U* = 28, N1 = 8, N2 = 7	0.07 (0.03; 0.15)*
AUC_0–∞_	3.61 ± 1.12 (31.0)	1.19 ± 1.07 (89.8)	0.0009 *t*_13_ = − 4.26	0.16 (0.06; 0.45)
*4-OH ATO*				
*C*_max_ (ng/mL)	3.18 ± 1.78 (55.9)	2.34 ± 1.27 (54.2)	0.3184 *t*_13_ = − 1.04	0.72 (0.42; 1.24)
AUC_0–*t*_ (ng × h/mL)	14.63 ± 5.57 (38.1)	8.04 ± 2.80 (34.9)	0.0143 *t*_13_ = − 2.82	0.55 (0.38; 0.79)
AUC_0–∞_ (ng × h/mL)	18.32 (12.89; 24.73)	9.60 (8.11; 11.37)	0.0046 *U* = 31, N1 = 8, N2 = 7	0.55 (0.41; 0.73)*
*4-OH ATO/ATO*				
*C*_max_	0.04 ± 0.02 (40.7)	0.04 ± 0.03 (78.0)	0.4781 *t*_13_ = − 0.73	0.64 (0.33; 1.23)
AUC_0–*t*_	0.08 ± 0.02 (28.3)	0.04 ± 0.02 (59.1)	0.0084 *t*_13_ = − 3.10	0.45 (0.28; 0.73)
AUC_0–∞_	0.10 ± 0.04 (38.1)	0.05 ± 0.03 (52.4)	0.0124 *t*_13_ = − 2.90	0.45 (0.27; 0.73)

Data from the control group (III_ATO_) was adopted from our previous study [12]

*REG* regorafenib, *ATO* atorvastatin, *M-2* regorafenib *N*-oxide, *M-5* *N*-desmethyl regorafenib *N*-oxide, *2-OH* *ATO* orthohydroxy atorvastatin, *4-OH* parahydroxy atorvastatin, *n* number of animals, *CI* confidence interval, *C*_*max*_ the maximum plasma concentration, *AUC*_*0*–*t*_ the area under the plasma concentration–time curve from zero to the time of the last measurable concentration, *AUC*_*0*–*∞*_ the area under the plasma concentration–time curve from zero to infinity, *t*_*max*_ the time to the first occurrence of *C*_max_, *k*_*el*_ the elimination rate constant, *t*_*1/2*_ the elimination half-life, *Cl*/*F* the apparent plasma drug clearance, *V*_*d*_/*F* the apparent volume of distribution

*Non-parametric test. Arithmetic means and standard deviations (SD) are shown with coefficients of variation CV (%) in brackets for parametric test; median values with quartiles in case of non-parametric test

**Fig. 2:**
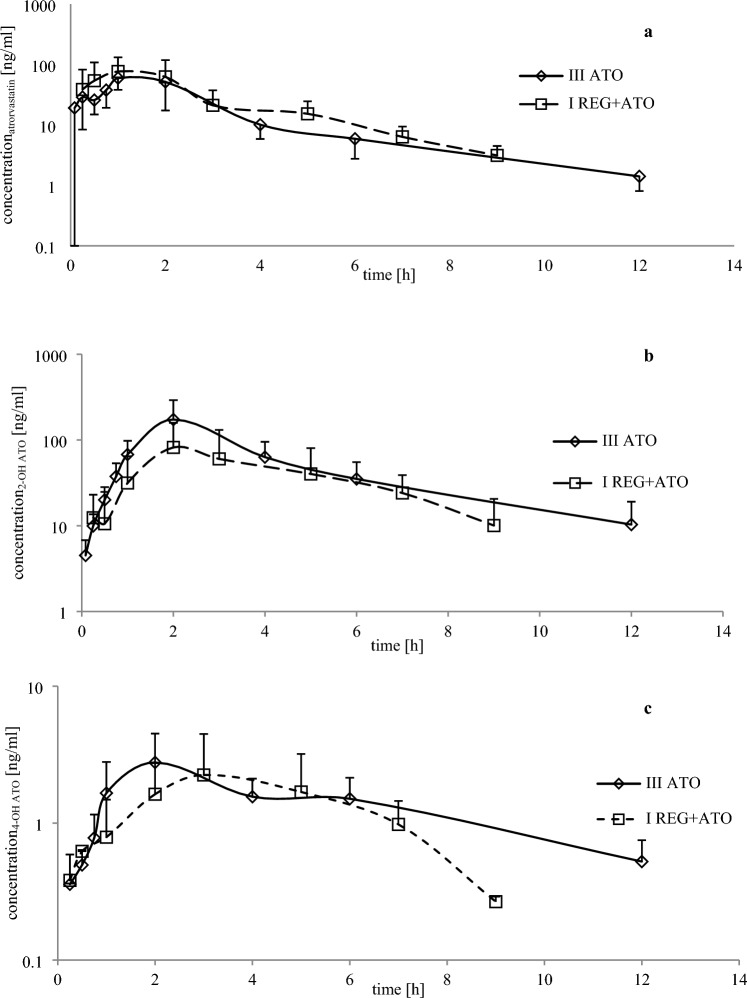
The ATO (**a**), 2-OH ATO (**b**), and 4-OH ATO (**c**) plasma concentration–time profiles in rats after a single oral dose of ATO 20 mg/kg b.w. (III_ATO_; *n* = 8) and REG 20 mg/kg b.w. + ATO 20 mg/kg b.w. (I_REG+ATO_; *n* = 8). The data regarding the control group were adopted from our previous study [12]. *REG* regorafenib, *ATO* atorvastatin, *M-2* regorafenib *N*-oxide, *M-5* *N*-desmethyl regorafenib *N*-oxide, *2-OH ATO* orthohydroxy atorvastatin, *4-OH* parahydroxy atorvastatin, *n* number of animals

In the presence of REG, the k_el_ of ATO increased significantly by 58.3%. There were no significant differences in other pharmacokinetic parameters of ATO between the III_ATO_ and I_REG+ATO_ groups (Table 3, Fig. 2a).

The exposure to 2-OH ATO was significantly lower in the presence of REG, as evidenced by lower values of AUC_0–*t*_ and AUC_0–∞_, which decreased by 86.9% and 67.3%, respectively (Table 3, Fig. 2b).

When ATO and REG were co-administered, AUC_0–*t*_ and AUC_0–∞_ of 4-OH ATO decreased by 45.0% and 46.8%, respectively (Table 3, Fig. 2c).

In the presence of REG, AUC_0–t_, AUC_0–∞_, as well as the 2-OH ATO/ATO, and 4-OH ATO/ATO ratios decreased by 87.0%, 67.0%, 50.0%, and 50.0%, respectively (Table 3).

The sum of the *C*_max_ for ATO, 2-OH ATO, and 4-OH ATO in the III_ATO_ group (251.14 ± 138.01 ng/mL; CV = 55.0) was greater (*p* = 0.3016; *G*_mean_ ratio = 0.76, and 90% CI = 0.46; 1.23) than in the I_REG+ATO_ group (170.27 ± 92.71 ng/mL; CV = 54.4), but these values were not statistically significant.

## Discussion

Our research aimed to assess the bilateral pharmacokinetic interactions of ATO, REG, and their active metabolites in healthy rats. We found that a single administration of ATO increased the exposure to REG and its active metabolites. REG, in turn, decreased the AUC of both metabolites of ATO. The ratio of AUC_in the presence of the perpetrator_/AUC_in the absence of the perpetrator_ was used to assess the significance of the interaction. Weak inhibitors of the CYP enzyme have been indicated to increase the AUC ratio by 1.25–1.99-fold, moderate inhibitors by 2–4.99-fold, and strong inhibitors by ≥ fivefold; weak inducers, in turn, were proven to reduce AUC by 20–49%, moderate inducers by 50–79%, and strong inducers by ≥ 80% [16–18]. The details of the observed interaction are discussed below.

### The influence of ATO on the pharmacokinetics of REG and M-2 and M-5 metabolites

Compared to REG alone, ATO significantly increased the exposure of REG and its two metabolites, M-2 and M-5 (Table 2). The increase in the *C*_max_, AUC_0–*t*_, and AUC_0–∞_ of REG (Table 2) in the presence of ATO (the perpetrator) may have been caused by the ATO-mediated inhibition of the CYP3A4 isoenzyme in the small intestine and liver [11]. In turn, the differences in REG and M-5 exposure can be explained by increased absorption. The parallel elimination of REG and M-5 in the I_REG+ATO_ and III_ATO_ groups indicates markedly better absorption of both compounds (Fig. 1a–c). ATO-mediated inhibition of the CYP3A4 isoenzyme in the liver may also reduce M-2 metabolism and increase its plasma concentration, as M-2 is further metabolized by CYP3A4 [5, 6, 19]. In rats, the total CYP content in the small intestine constitutes only 6% of the liver content. Both in humans and rodents, the liver generates more M-2 than the small intestine. However, humans and primates generally produce more M-2 in the small intestine than rodents [19]. The values of the AUC_in the presence of perpetrator_/AUC_in the absence of perpetrator_ ratio seem to indicate that ATO was a moderate inhibitor [16, 17]. Several studies indicated ATO as a relatively weak inhibitor of CYP3A4 [11, 21]. Its IC_50_ (half-maximal inhibitory concentration) is 48.0 mmol/L, whereas the IC_50_ values of pravastatin and simvastatin are 14.1 mmol/L and 3.10 mmol/L, respectively [11, 22].

It is also important to consider the role of P-gp in the investigated interaction mechanism. Increased exposure to REG may have been caused by the inhibitory effect of ATO on the P-gp efflux pump in the small intestine [11, 20, 21]. The increase in the plasma *C*_max_ of M-2 and M-5 (which are both P-gp substrates) in the I_REG+ATO_ group, observed in our study, may also have been caused by the decrease of their renal elimination due to the inhibition of the renal P-gp by ATO [23]. Research on humans has shown that after a daily dose of REG exceeding 80 mg, M-2 and M-5 tend to accumulate in blood [24]. In our study, the single dose of REG administered to rats was 20 mg/kg b.w. Statins, including ATO, are known inhibitors of P-gp [11, 21, 23, 25], also in rodents [25]. In one example, the inhibition of P-gp by ATO increased imatinib and dasatinib exposure [26]. Moreover, the Cl/F of REG decreased by 72.7% in the presence of ATO, which may also have contributed to the increase in the *C*_max_, AUC_0–*t*_, and AUC_0–∞_ of REG.

The M-2/REG ratios for *C*_max_, AUC_0–*t*_, and AUC_0–∞_ were similar in both groups, whereas the M-5/REG ratios were significantly higher in the presence of ATO (*p* < 0.05) (Table 2). The sum of the *C*_max_ of REG, M-2, and M-5 was also significantly higher (*p* = 0.0033) in the I_REG+ATO_ group. When analyzing such a complex interaction, the contribution of metabolites to drug exposure must also be considered. Zopf et al. [27] investigated the plasma pharmacokinetics of REG and M-2 and M-5 in female NMRI Foxn1 nu/nu mice after repeated oral administration of 10 mg/kg of REG for 5 consecutive days. The researchers found that the *C*_max_ values of M-2 and M-5 accounted for 15.17% and 1.27% of the REG *C*_max_, respectively. In our study, after a single dose of REG (20 mg/kg), the M-2 and M-5 *C*_max_ values were very similar, respectively, 15.28% and 1.17% in the REG group, and 24.14% and 2.12% in the I_REG+ATO_ group. This indicates that, even after a single dose of REG, M-2 remains its dominant metabolite in rodents [6, 19, 27]. Before drug dosage modification, the exposure of the active metabolite to the parent drug must be considered. If it is greater than 10%, it is necessary to consider the strength of its pharmacological effect. Then, if this effect accounts for less than 50% of total drug activity, its role in drug interactions can be omitted [18]. When a single dose of REG is administered to humans, peak plasma concentrations of M-2 and M-5 are significantly lower than those of the principal compound. In contrast, at a steady state, concentrations of both REG and its metabolites are comparable [5]. Moreover, in vitro*,* both metabolites have similar pharmacological activity to REG [19]. Fukudo et al. [28] found that the right blood concentration of REG and its metabolites was important for the progression-free survival (PSF) of patients with advanced metastatic colorectal cancer, gastrointestinal stromal tumor, and hepatocellular carcinoma. However, larger, randomized trials are needed to confirm these observations.

An alternative statin could be proposed, assuming the significance of the above interaction with ATO. Currently, two statins are commonly used in the pharmacotherapy of dyslipidemia: ATO and rosuvastatin. Therefore, identification of their potential interactions is of great practical importance, as was highlighted in the recommendations of both the European Society of Cardiology (ESC) and the European Atherosclerosis Society (EAS) [29]. As rosuvastatin does not cause as many interactions as ATO, it should be considered the statin of choice in patients at risk of such complications. However, a simple replacement of these statins is not always recommended, e.g., in patients with renal failure. The PLANET and SATURN clinical trials showed that rosuvastatin caused more cases of hematuria, proteinuria, and kidney failure than ATO. Hence, ATO has been identified as the more renoprotective statin, and is dedicated to patients with renal failure [29, 30].

### The influence of REG on the pharmacokinetics of ATO, 2-OH ATO, and 4-OH ATO

Our study showed that a single dose of REG did not inhibit the elimination of ATO. The only significant increase between III_ATO_ and I_REG+ATO_ (by 58.3%) was observed for the *k*_el_ of ATO. There were no significant differences in the other pharmacokinetic parameters of ATO between these groups. 2-OH ATO exposure in the experimental group was significantly lower than in the control group. A similar effect was observed for the 4-OH ATO metabolite (Table 3, Fig. 2). Decreased exposure to 2-OH ATO and 4-OH ATO metabolites may have been caused by a shift in metabolism towards lactone forms. However, these metabolites were not analyzed in our study, which is a significant limitation. About 70% of the HMG-CoA inhibitory activity in the systemic circulation is attributed to active metabolites. 2-OH ATO and 4-OH ATO also inhibit HMG-CoA reductase, with an effect comparable to the primary drug [31]. The sum of the *C*_max_ of ATO, 2-OH ATO, and 4-OH ATO in the III_ATO_ group (251.14 ± 138.01 ng/mL) was greater than in the I_REG+ATO_ group (170.27 ± 92.71 ng/mL). Nonetheless, the obtained differences were not statistically significant (*p* = 0.3016).

A number of studies reported effectiveness of combination therapy involving statins, such as ATO [32, 33] and anticancer drugs [32–35]. The combination of ATO with tyrosine kinase inhibitors (TKIs) such as gefitinib, erlotinib [36] or sorafenib resulted in enhanced antitumor activity against TKI-resistant hepatocellular carcinoma and non-small cell lung cancer. ATO may also reduce the incidence and severity of cisplatin-induced hearing loss [37]. These reports underline the importance of evaluating interactions between ATO and REG. Yuan et al. [38] found that the combination of rosuvastatin and REG had a synergistic effect, which may be a new standard of colorectal cancer treatment.

Summarizing, ATO and REG interactions observed in animals in this study may have clinical implications. Hence, considering the possibility of REG-ATO interaction in clinical practice may lead to improvements in treatment strategies. Determining optimal dosage regimens of both drugs for patients with advanced gastrointestinal cancer may reduce the risk of adverse reactions dependent on the concentration of REG (e.g., hyperbilirubinemia and liver function disorders) and its metabolites (e.g., hypertension and toxic erythema) in the blood. So far, a consensus has yet to be reached regarding the therapeutic scope of REG in plasma. Therefore, it is impossible to extrapolate the results of our study directly to cancer patients without conducting extensive and detailed clinical tests.

### Limitations of the study

There are several limitations that must be considered when drawing conclusions from this study. Firstly, the pharmacokinetics of REG and ATO in rats may differ from humans. Thus, the differences among species regarding CYP450 should be considered. However, the activity of CYP3A in male rats is closest to that in humans, with CYP3A1, the rat orthologue of CYP3A4, exhibiting 73% amino acid homology with its human counterpart. Secondly, the study was conducted on healthy animals without cancer and hyperlipidemia. However, such experimental design eliminated the potential influence of comorbidities on inter-individual variability. Another limitation is that, due to the requirement to minimize the number of control animals, the pharmacokinetic data for the control group (III_ATO_), receiving ATO alone, were adopted from our earlier study. Because the study design and experimental conditions were identical in both experiments, we assumed that this constraint should not influence the results and data interpretation.

### Conclusions

This animal model study showed a significant pharmacokinetic interaction between REG and ATO. While this interaction may be clinically significant, this needs to be confirmed in clinical trials involving cancer patients.

## Data Availability

The authors declare that the data supporting the findings of this study are available within the paper and its Supplementary Information files. Should any raw data files be needed in another format they are available from the corresponding author upon reasonable request.

## Abbreviations

2-OH ATO: Orthohydroxy atorvastatin
4-OH ATO: Parahydroxy atorvastatin
ATO: Atorvastatin
AUC_0–∞_: The area under the plasma concentration–time curve from zero to the time of the last measurable concentration
AUC_0–__*t*_: The area under the plasma concentration–time curve from zero to infinity
BCRP: Breast Cancer Resistance Protein
CI: Confidence interval
CL/F: The apparent plasma drug clearance
*C*_max_: The maximum plasma concentration
CV: Coefficient of variation
CYP3A4: Cytochrome P450 3A4
CRC: Colorectal cancer
DMSO: Dimethyl sulfoxide
FGFR: Fibroblast growth factor receptor
GIST: Gastrointestinal stromal tumor
HCC: Hepatocellular carcionoma
HMG-CoA: 3-Hydroxy-3-methylglutaryl-coenzyme A
*k*_el_: The elimination rate constant
LLOQ: Lower limit of quantification
M-2: Regorafenib *N*-oxide
M-5: *N*-Desmethyl regorafenib *N*-oxide
MeOH: Methanol
*n*: Number of animals
NCA: Non-compartmental analysis
PDGFR: Platelet-derived growth factor receptor
P-gp: P-glycoprotein
PK: Pharmacokinetics
*r*: Correlation coefficient
*R*^2^: Coefficient of determination
REG: Regorafenib
RET: Rearranged during transfection (*oncogene*)
SD: Standard deviation
TIE2: Angiopoietin
*t*_max_: The time to the first occurrence of *C*_max_
*t*_1/2_: The elimination half-life
UGT1A9: UDP-glucuronosyltransferase 1–9
UPLC-MS/MS: Ultra-performance liquid chromatography-tandem mass spectrometry
VEGF: Vascular endothelial growth factor
*V*_d_/*F*: The apparent volume of distribution
